# Mesenchymal stem cells prevent the progression of diabetic nephropathy by improving mitochondrial function in tubular epithelial cells

**DOI:** 10.1038/s12276-019-0268-5

**Published:** 2019-07-09

**Authors:** Seung Eun Lee, Jung Eun Jang, Hyun Sik Kim, Min Kyo Jung, Myoung Seok Ko, Mi-Ok Kim, Hye Sun Park, Wonil Oh, Soo Jin Choi, Hye Jin Jin, Sang-Yeob Kim, Yun Jae Kim, Seong Who Kim, Min Kyung Kim, Chang Ohk Sung, Chan-Gi Pack, Ki-Up Lee, Eun Hee Koh

**Affiliations:** 1 Department of Internal Medicine, University of Ulsan College of Medicine, Seoul, Korea; 2 Asan Institute for Life Sciences, University of Ulsan College of Medicine, Seoul, Korea; 3 Biomedical Research Institute, MEDIPOST Co., Ltd., Gyeonggi‑do, Korea; 4 Department of Convergence Medicine, University of Ulsan College of Medicine, Seoul, Korea; 5 Department of Biochemistry and Molecular Biology, University of Ulsan College of Medicine, Seoul, Korea; 6 Department of Pathology, University of Ulsan College of Medicine, Seoul, Korea; 7 Present Address: Department of Internal Medicine, Dongguk University Ilsan Hospital, Dongguk University College of Medicine, Goyang, Korea; 8 Present Address: Department of Internal Medicine, Haeundae Paik Hospital, Inje University College of Medicine, Busan, Korea

**Keywords:** Diabetes complications, Mechanisms of disease

## Abstract

The administration of mesenchymal stem cells (MSCs) was shown to attenuate overt as well as early diabetic nephropathy in rodents, but the underlying mechanism of this beneficial effect is largely unknown. Inflammation and mitochondrial dysfunction are major pathogenic factors in diabetic nephropathy. In this study, we found that the repeated administration of MSCs prevents albuminuria and injury to tubular epithelial cells (TECs), an important element in the progression of diabetic nephropathy, by improving mitochondrial function. The expression of M1 macrophage markers was significantly increased in diabetic kidneys compared with that in control kidneys. Interestingly, the expression of arginase-1 (*Arg1)*, an important M2 macrophage marker, was reduced in diabetic kidneys and increased by MSC treatment. In cultured TECs, conditioned media from lipopolysaccharide-activated macrophages reduced peroxisomal proliferator-activated receptor gamma coactivator 1α (*Pgc1a*) expression and impaired mitochondrial function. The coculture of macrophages with MSCs increased and decreased the expression of *Arg1* and M1 markers, respectively. Treatment with conditioned media from cocultured macrophages prevented activated macrophage-induced mitochondrial dysfunction in TECs. In the absence of MSC coculture, *Arg1* overexpression in macrophages reversed *Pgc1a* suppression in TECs. These observations suggest that MSCs prevent the progression of diabetic nephropathy by reversing mitochondrial dysfunction in TECs via the induction of *Arg1* in macrophages.

## Kidney disease: stem cells to the rescue

Stem cells can halt the progression of kidney damage owing to diabetes by reducing inflammation and improving energy production in kidney cells. Eun Hee Koh at the University of Ulsan College of Medicine in Seoul, South Korea, and colleagues found that adult stem cells, known as mesenchymal stem cells (MSCs), derived from human umbilical cord blood had a protective effect on the kidneys of diabetic mice. Repeated administration of MSCs prevented the recruitment of pro-inflammatory cells into the kidney and increased the levels of arginase-1, a marker of cells with anti-inflammatory activity. Experiments in cells showed that MSCs stimulated the production of arginase-1 in that, in turn, were able to increase the production and activity of mitochondria in kidney cells. This study confirms an important role for MSCs in organ repair.

## Introduction

Diabetic nephropathy is the leading cause of end-stage renal disease worldwide^1^. The results from animal studies have shown that xenogenic (human)^2,3^ as well as allogenic mesenchymal stem cells (MSCs)^4^ favorably modulate diabetic nephropathy. In addition to their primary prevention of diabetic nephropathy^5^, the repeated administration of MSCs prevents progression to overt albuminuria or advanced diabetic nephropathy^3,6^. However, the underlying mechanisms of these beneficial effects have not been adequately elucidated.

Mitochondrial dysfunction, which leads to oxidative stress, cell death, metabolic dysfunction, and inflammation^7^, is a major pathogenic factor in diabetic nephropathy^8^. Accordingly, the restoration of whole-body mitochondrial function by exercise, caloric restriction, and drugs protected kidney function in several animal models of nephropathy^9–12^. In addition, previous studies have shown that stem cells enhance mitochondrial function and bioenergetics in various models of human diseases^13,14^.

Mitochondria are rich in podocytes and tubular epithelial cells (TECs)^15^. Although podocyte injury is the primary source of lesion of diabetic nephropathy, recent studies proposed that proximal tubular cells have a major role in both the initiation and progression of diabetic nephropathy^16^. Indeed, the progression of diabetic nephropathy correlates well with tubular degeneration and interstitial fibrosis^17^. Interestingly, a recent study found that the renoprotective effects of sodium-glucose cotransporter 2 inhibitors can be attributed to an improvement in mitochondrial function in renal tubules^18^. Hence, we investigated whether MSC therapy prevents the progression of diabetic nephropathy by modulating mitochondrial damage in TECs.

Macrophage infiltration is a characteristic feature of diabetic nephropathy^19^, which is associated with renal fibrosis^20^. A growing body of evidence suggests that the pro-inflammatory cytokines produced by activated macrophages contribute to the development of progressive renal diseases^21^. In addition to glucolipotoxicity^22^, recent studies have suggested that activated macrophages contribute to renal inflammation and fibrosis^23^. MSCs are well known for their immunomodulatory functions^24^. Recent studies, including ours^25,26^, have shown that the inflammatory cytokines produced by activated macrophages impair mitochondrial function in adipocytes. Hence, we hypothesized that MSC treatment may improve mitochondrial function in the diabetic kidney via the suppression of activated macrophages. In this study, we found that the administration of human umbilical cord blood-derived MSCs prevents the progression of diabetic nephropathy by inducing arginase-1 (*Arg1*), a key M2 marker that inhibits M1 polarization^27,28^, in macrophages to improve mitochondrial biogenesis and function in TECs.

## Materials and methods

### Animals

All animal-based and experimental protocols were approved by the Institutional Animal Care and Use Committee of the Asan Institute for Life Sciences, Seoul, Korea. Eight-week-old male CD1 mice (Orient Bio Inc., Korea) were intraperitoneally injected with streptozotocin (STZ, 80 mg/kg; Sigma-Aldrich, St. Louis, MO) dissolved in citrate buffer (pH 4.5) or citrate buffer alone (control) for 3 days. Five weeks after the induction of diabetes, human umbilical cord blood-derived MSCs were administered to mice three times (Supplementary Fig. 1). Human MSCs were prepared as described previously^29^. The mice were killed 24 weeks after STZ injection. Confocal microscopy using frozen sections of kidney tissue was performed to evaluate the localization of MSCs after tail vein injection in diabetic mice.

### Nephrectomy procedures

To establish a diabetic mouse model with advanced nephropathy, we performed unilateral nephrectomy in eight-week-old mice anesthetized with pentobarbital sodium (Entobar®; 30 mg/kg, i.p.; Hanlim Pharmaceuticals, Seoul, Korea). The mice were allowed to recover for 1 week and were then injected with STZ (80 mg/kg; sigma) dissolved in citrate buffer (pH 4.5) or citrate buffer alone (control) for 3 consecutive days. Sixteen weeks after the STZ injection, the mice were killed and analyzed.

### Biochemistry

Urinary albumin levels were measured using a mouse albumin enzyme-linked immunosorbent assay kit (Immunology Consultants Laboratory Inc., Newberg, OR), and urinary creatinine levels were measured using a colorimetric detection kit (cat. #907-030) (Assay Designs Inc., Ann Arbor, MI). The urinary albumin-to-creatinine ratio (UACR) was defined as the amount of urinary albumin divided by the amount of urinary creatinine (mcg/mg). Blood samples were collected after 12 h of fasting. The plasma glucose level was determined using the glucose and lactate analyzer YSI 2300 (Yellow Springs Instruments, Yellow Springs, OH). Plasma blood urea nitrogen (BUN) and creatinine levels were measured using a Hitachi 7180 chemical analyzer (Hitachi Ltd., Tokyo, Japan).

### Histological analysis

Tissues were fixed in 10% formalin for dehydration, embedded in paraffin, and sectioned. Renal tissues were stained with hematoxylin and eosin. Furthermore, tissue sections were stained with periodic acid-Schiff base (PAS) and Masson’s trichrome (MT) stain to evaluate changes in the glomerular basement membrane and fibrotic changes, respectively.

### Gene expression analysis

Total RNA was isolated using TRIzol reagent (Invitrogen, Carlsbad, CA), and 1 µg of each sample was reverse-transcribed into cDNA with random primers using a Reverse Aid M-MuLV reverse transcription kit (Fermentas, Hanover, MD). The relative mRNA expression levels were determined using real-time PCR and gene-specific primers with the 7500 Fast Real-Time PCR System (Applied Biosystems, Foster City, CA) with a SYBR Green PCR kit (Applied Biosystems). The primers used are shown in Supplementary Table 1. The expression levels of each gene were normalized to those of *Tbp1*, a housekeeping gene.

### Immunostaining

The engraftment of infused MSCs derived from umbilical cord blood was analyzed using immunofluorescence staining of human β2-microglobulin (Abcam, Cambridge, UK) and visualized using an Alexa 488-labeled secondary antibody. Nuclei were counterstained with Hoechst 33342 (Invitrogen). Images were acquired using a ZEISS LSM 800 confocal microscope (Carl Zeiss, Munich, Germany). MSCs stained with human β2-microglobulin were detected in kidney tissues (Supplementary Fig. 2).

### Cell culture

RAW264.7 cells, a murine macrophage cell line, were cultured in Dulbecco’s modified Eagle’s medium. For coculture experiments, MSCs were added at a ratio of 1:2 (MSCs: RAW264.7 cells). After 24 h of incubation, lipopolysaccharides (LPSs) were added to the RAW264.7 cells with or without MSCs at a final concentration of 10 ng/ml. Supernatants were collected 24 h after LPS stimulation, and the conditioned media were transferred to HK-2 cells (1.5 × 10^5^ cells per well) for 24 h. The remaining RAW264.7 cells after supernatant collection were harvested for phenotypic analyses (Supplementary Fig. 3). To evaluate the effect of interleukin-10 (IL-10) on *Arg1* expression, RAW264.7 cells were pretreated with or without IL-10 (PeproTech, Rocky Hill, NJ) at a concentration of 1 ng/ml or 10 ng/ml for 6 h. Next, RAW264.7 cells were treated with LPS for 24 h. The cells remaining after supernatant collection were harvested for analysis.

### Measurement of mitochondrial ROS production

Mitochondria-specific reactive oxygen species (ROS) generation was measured using MitoSOX Red fluorescent dye (Molecular Probes, Eugene, OR) according to the manufacturer’s instructions. After serum starvation for 24 h, cultured cells grown on collagen type IV-coated coverslips were treated with conditioned media from RAW264.7 cells for the indicated time points and stained with MitoSOX Red (5 μm) for 20 min at 37 °C. Cellular fluorescence was measured using flow cytometry with excitation at 510 nm and emission at 580 nm.

### Visualization of mitochondria using MitoTracker Red

Cells grown on coverslips were incubated with MitoTracker Red (100 nm; Molecular Probes) and maintained in phosphate-buffered saline (PBS) (Welgene, Daegu, Korea). Confocal images were captured using a Zeiss LSM 510 META microscope (Carl Zeiss).

### Western blotting

Cells were lysed in buffer containing 20 mm Tris-HCl (pH 7.4), 1 mm EDTA, 140 mm NaCl, 1% NP-40, 1 mm Na_3_VO_4_, 1 mm phenylmethylsulfonyl fluoride, 50 mm NaF, and 10 μg/ml aprotinin. Proteins (10–20 μg) were resolved using sodium dodecyl sulfate polyacrylamide gel electrophoresis, transferred to nitrocellulose membranes, and immunoblotted with specific antibodies. Antibodies against PGC-1α and COX-IV were obtained from Cell Signaling Technology (Danvers, MA). An antibody against mtTFA was purchased from Santa Cruz Biotechnology (Santa Cruz, CA). The band intensities were normalized to that of GADPH (Cell Signaling Technology) to correct for variations in sample loading.

### Measurement of cellular respiration and glycolysis rates

The oxygen consumption rate (OCR) and the extracellular acidification rate (ECAR) in HK-2 cells were measured using an XF24 instrument (Seahorse Bioscience, North Billerica, MA) according to the manufacturer’s specifications^30^.

### Overexpression of arginase-1 and arginase-2 in macrophages

Full-length mouse *Arg1* and *Arg2* cDNA were cloned using RT-PCR and subcloned into the pCDH-MCS lentiviral vector (System Bioscience, Palo Alto, CA). The *Arg1*, *Arg2* and control lentiviruses were produced by transfecting Lenti-X 293 T cells (Clontech, Mountain View, CA) with the pCDH-*Arg1*, pCDH-*Arg2*, pCDH-MCS (control), and packaging (pCMV-VSV-G, pMDLg/pRRE, and pRSV-Rev; Addgene) plasmids using Lipofectamine 3000 (Invitrogen). Infectious lentiviruses were harvested 48 h post transfection and then used to infect RAW264.7 cells with 8 μg/ml polybrene (Sigma-Aldrich).

### Nitric oxide measurement

NO production was estimated using an NO assay kit (ENZO Life Sciences, Lausen, Switzerland). Supernatants (25 μl) from each RAW264.7 cell culture were processed according to the manufacturer’s instructions.

### Electron microscopy

After removal of the kidneys, the tissues were immediately diced into 1 mm^3^ pieces and fixed with 2.5% glutaraldehyde and 2% paraformaldehyde in sodium cacodylate buffer (pH 7.2) at 4 °C. Tissue specimens were then postfixed in 1% osmium tetraoxide (OsO_4_) containing 1.5% potassium ferrocyanide for 30 min at 4 °C. The fixed tissues were dehydrated using an ethanol series (50%, 60%, 70%, 80%, 90%, and 100%) for 20 min at each concentration. The tissues were subsequently transferred to propylene oxide (Sigma-Aldrich) and embedding media (Spurr’s Kit; Electron Microscopy Science, Hatfield, PA). After impregnating pure resin, the tissue specimens were embedded in the same resin mixture and sectioned (60–70 nm) using an ultramicrotome (Leica Microsystems GmbH, Vienna, Austria). Then, they were double-stained with 2% uranyl acetate for 20 min and lead citrate for 10 min. The sections were then observed using a Hitachi H7600 transmission electron microscope (TEM) (Hitachi Ltd.) at 80 kV. The number of abnormal mitochondria was determined by randomly capturing 20 TEM micrographs per sample (image area = 8.5 µm^2^, × 2000 magnification).

### Isolation and culture of adipose tissue-derived MSCs (AD-MSCs)

Adipose tissue was isolated from women undergoing liposuction. It was treated with 0.075% collagenase type I (Worthington Biochemical Corporation, Lakewood, NJ) in PBS (Amresco LLC, Solon, OH) for 30 min at 37 °C with gentle shaking. The collagenase was inactivated by the addition of an equal volume of alpha-minimum essential medium (α-MEM) (Gibco, Carlsbad, CA) supplemented with 10% fetal bovine serum (FBS) (Gibco) and a 100 μg/ml penicillin and streptomycin solution (Gibco). The suspension was then centrifuged at 3,000 rpm for 10 min to separate the floating adipocytes and remove the debris. The cells were plated and incubated in culture medium (α-MEM supplemented with 10% FBS and a 100 μg/ml penicillin and streptomycin solution) in a T175 flask. After 48 h, the nonadherent cells were removed and washed with PBS. Spindle-shaped cells were obtained by day 4 of culture. Subculture was performed when the cells reached 80% confluence. These cells were then incubated at 37 °C in a humidified atmosphere containing 5% CO_2_.

### Statistical analysis

Data were expressed as the mean ± SEM, and *P* values were calculated using two-tailed Student’s *t* test for the pairwise comparison of variables and one-way analysis of variance for the comparison of multiple variables. Bonferroni correction was applied for comparisons of multiple variables. Statistical analyses were conducted using SPSS version 20 (SPSS Inc., Chicago, IL). A *P* value < 0.05 indicated statistical significance.

## Results

### MSCs reversed albuminuria and prevented the progression of diabetic nephropathy

**Fig. 1:**
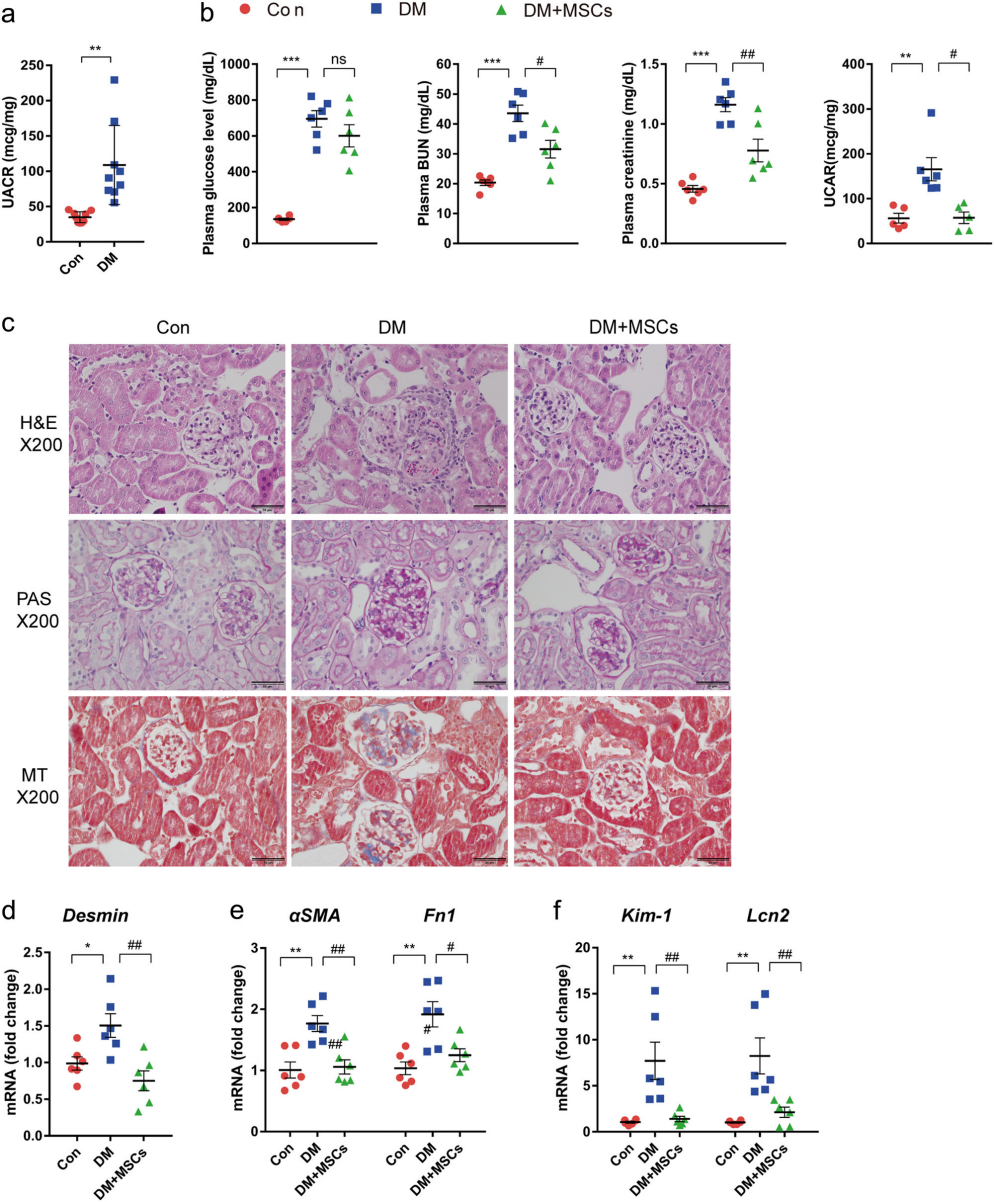
MSC treatment prevented the progression of diabetic nephropathy. Diabetic mice were treated with vehicle or MSCs 5, 9, and 13 weeks after STZ injection and sacrificed at 24 weeks. **a** Albuminuria, defined by the UACR, was markedly increased in diabetic mice 4 weeks after STZ injection. **b** Changes in plasma glucose, BUN, creatinine, and UACR levels in diabetic mice treated with or without MSCs. **c** Representative H&E, PAS, and MT staining of kidney sections. Scale bars, 50 µm. **d**–**f** MSC treatment prevented renal damage in diabetic mice. The mRNA levels of *Desmin*, a marker of podocyte injury **d**; *α-SMA*, and *Fn1*, markers of fibrosis **e**; and *Kim-1* and *Lcn2*, markers of tubular epithelial injury **f**. Con, control non-diabetic CD1 mice; DM, diabetic CD1 mice; DM + MSCs, diabetic CD1 mice with MSC treatment. Data show the means ± SEMs of 6–9 mice. ^*^P < 0.05, ^**^P < 0.01, and ^***^P < 0.001 versus Con; ^#^*P* < 0.05, and ^##^*P* < 0.01 versus DM

Diabetic mice presented a significantly increased urinary albumin excretion rate, as scored by the UACR, compared with that in control mice 4 weeks after STZ administration (Fig. 1a). To evaluate the effect of MSCs on the progression of diabetic nephropathy, we injected mice with MSCs three times (5, 9, and 13 weeks after STZ injection) after the establishment of albuminuria (Supplementary Fig. 1). The mice were killed 24 weeks after STZ induction, and the blood, urine, and kidneys were harvested for analysis. Immunostaining of human β2-microglobulin revealed the engraftment of MSCs in the diabetic kidney 11 weeks after the last MSC injection (Supplementary Fig. 2).

As expected, diabetic mice showed increased plasma levels of BUN, creatinine, and UACR (Fig. 1b). Although the administration of MSCs did not reduce the plasma glucose level, it significantly lowered the plasma BUN, creatinine, and UACR levels (Fig. 1b).

Histological examination of diabetic mice showed glomerular mesangial expansion, matrix accumulation in the mesangial area, a thickened basement membrane, and focal glomerular sclerosis (Fig. 1c). However, advanced histological changes in the tubulointerstitial area, such as atrophic tubules, interstitial inflammation, and fibrosis, were not observed even in the diabetic mice that had undergone unilateral nephrectomy, which was shown to accelerate the progression of diabetic nephropathy^31^ (data not shown). The expression of *Desmin*, a marker of podocyte injury^32^, was elevated in diabetic mice compared with its expression in control mice (Fig. 1d). Although advanced histological changes in the tubulointerstitial area were not observed, the mRNA levels of genes encoding alpha smooth muscle actin (*αSMA*) and fibronectin (*Fn1*) (Fig. 1e), markers of epithelial-to-mesenchymal transition and fibrosis^33^, respectively, were elevated in the kidneys of diabetic mice. The levels of kidney injury molecule-1 (*Kim-1*) and lipocalin 2 (*Lcn2*), sensitive markers of early tubular injury^34,35^, were also significantly increased in diabetic mice (Fig. 1f). Treatment with MSCs ameliorated all the changes observed in diabetic mice (Fig. 1b–f).

### MSCs reduced macrophage infiltration and increased *Arg1* expression in diabetic kidneys

**Fig. 2:**
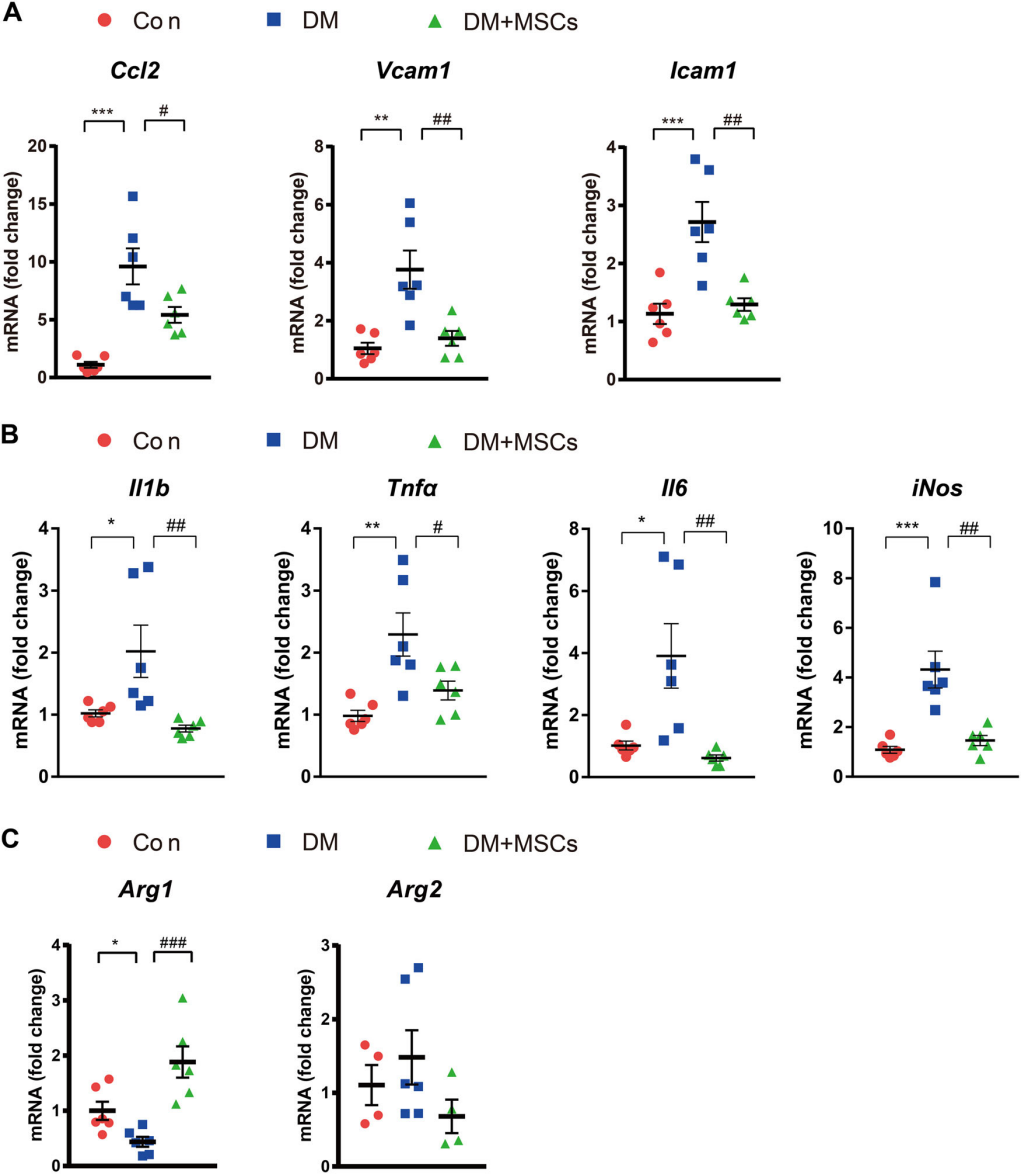
MSC treatment decreased and increased the expression of M1 macrophages and *Arg1*, respectively, in diabetic kidneys. **a** Real-time RT-PCR analysis of *Ccl2, Vcam1*, and *Icam1* expression in the kidneys. **b** MSC treatment decreased the expression level of M1 macrophage markers. **c** MSC treatment increased *Arg1* expression, whereas it did not alter *Arg2* expression. Con, non-diabetic CD1 mice; DM, diabetic CD1 mice; DM + MSCs, diabetic CD1 mice with MSC treatment. Data show the means ± SEMs of six mice. ^*^P < 0.05, ^**^P < 0.01, and ^***^P < 0.001 versus Con; ^#^*P* < 0.05, ^##^*P* < 0.01, and ^###^*P* < 0.001 versus DM

Treatment with MSCs prevented the increase in the expression of markers associated with macrophage recruitment, including the C-C motif chemokine ligand 2 (*Ccl2*), vascular cell adhesion molecule-1 (*Vcam1*), and intercellular adhesion molecule-1 (*Icam1*), in the kidneys of diabetic mice (Fig. 2a).

At least two distinct populations of macrophages infiltrate the diabetic kidney, namely, pro-inflammatory (M1) and anti-inflammatory (M2) macrophages. M1 macrophages cause tissue damage and inhibit cell proliferation, whereas M2 macrophages promote cell proliferation and tissue repair^27^. In our study, the mRNA levels of the M1 markers interleukin-1β (*Il1b*), tumor necrosis factor-alpha (*Tnfα*), interleukin-6 (*Il6*), and *iNos* were significantly increased in diabetic kidneys (Fig. 2b).

Among the M1 and M2 macrophage markers, inducible nitric oxide synthase (iNOS) and arginase are the most important molecules responsible for the activity of M1 and M2 macrophages, respectively^27,28^. M1 macrophages express iNOS, which metabolizes arginine to nitric oxide (NO) and citrulline. M2 macrophages are characterized by the expression of arginase, which hydrolyzes arginine to ornithine and urea. Thus, the arginase pathway limits arginine availability for NO synthesis, and ornithine itself can further feed into the important downstream polyamine and proline synthesis pathways, which are important for cellular proliferation and tissue repair^27,36^.

In our study, the expression of *Arg1* was significantly decreased in diabetic mice (Fig. 2c). In addition, MSC treatment significantly decreased M1 macrophage markers and increased *Arg1* expression (Fig. 2c), suggesting that increased *Arg1* expression is responsible for the anti-inflammatory action of MSCs.

The arginase gene expresses two isoforms, *Arg1* and *Arg2*. Of the two, *Arg2* is the dominant isoform in the kidney^37^. However, the expression level of *Arg2* was not significantly different between the groups (Fig. 2c).

### Coculture with MSCs increased *Arg1* expression in macrophages and suppressed M1 polarization

We next examined whether MSC coculture increased *Arg1* expression in cultured macrophages. To reproduce the local inflammatory condition of the kidney observed in diabetic nephropathy, we used LPSs. LPSs are fat-soluble outer membrane components of gram-negative bacteria that link many human diseases with inflammation^38^. In particular, high serum LPS activity has been associated with the progression of diabetic nephropathy in patients with type 1 diabetes mellitus^39^.

**Fig. 3:**
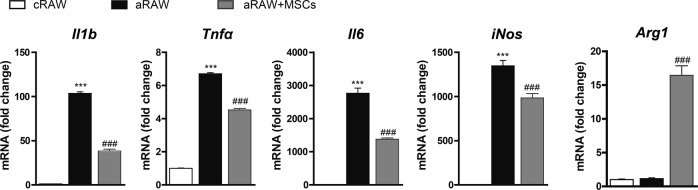
Coculture of RAW264.7 macrophages with MSCs suppressed LPS-induced M1 macrophage polarization and increased *Arg1* expression. MSCs were added to RAW264.7 cells at a ratio of 1:2 (MSCs: RAW264.7 ratio). After 24 h of incubation, LPSs were added to the cells at a final concentration of 10 ng/ml. cRAW, untreated RAW264.7 cells; aRAW, LPS-treated RAW264.7 cells; aRAW + MSCs, LPS-treated RAW264.7 cells cocultured with MSCs. Data are presented as the means ± SEMs (*n* = 4). ^***^P < 0.001 versus control (cRAW); ^###^*P* < 0.001 versus LPS-treated RAW264.7 cells (aRAW)

MSCs are well known for their immunomodulatory functions, which are exerted via direct cell-to-cell contact, cytokine secretion, and/or a combination of these mechanisms^24^. Hence, we investigated whether MSCs can reduce the pro-inflammatory activation of LPS-treated RAW264.7 cells, a murine macrophage cell line, in vitro. As expected, LPS-treated RAW264.7 cells (aRAW; activated RAW264.7 cells) showed the increased expression of M1 markers compared with their expression in control RAW264.7 cells (cRAW). Coculture with MSCs (aRAW + MSCs) significantly decreased the expression of these markers compared with their expression in those cultured without MSCs (aRAW cells) (Fig. 3). In addition, coculture with MSCs significantly increased *Arg1* expression in RAW264.7 cells (Fig. 3).

### *Arg1* overexpression in macrophages suppressed the M1 polarization of macrophages

**Fig. 4:**
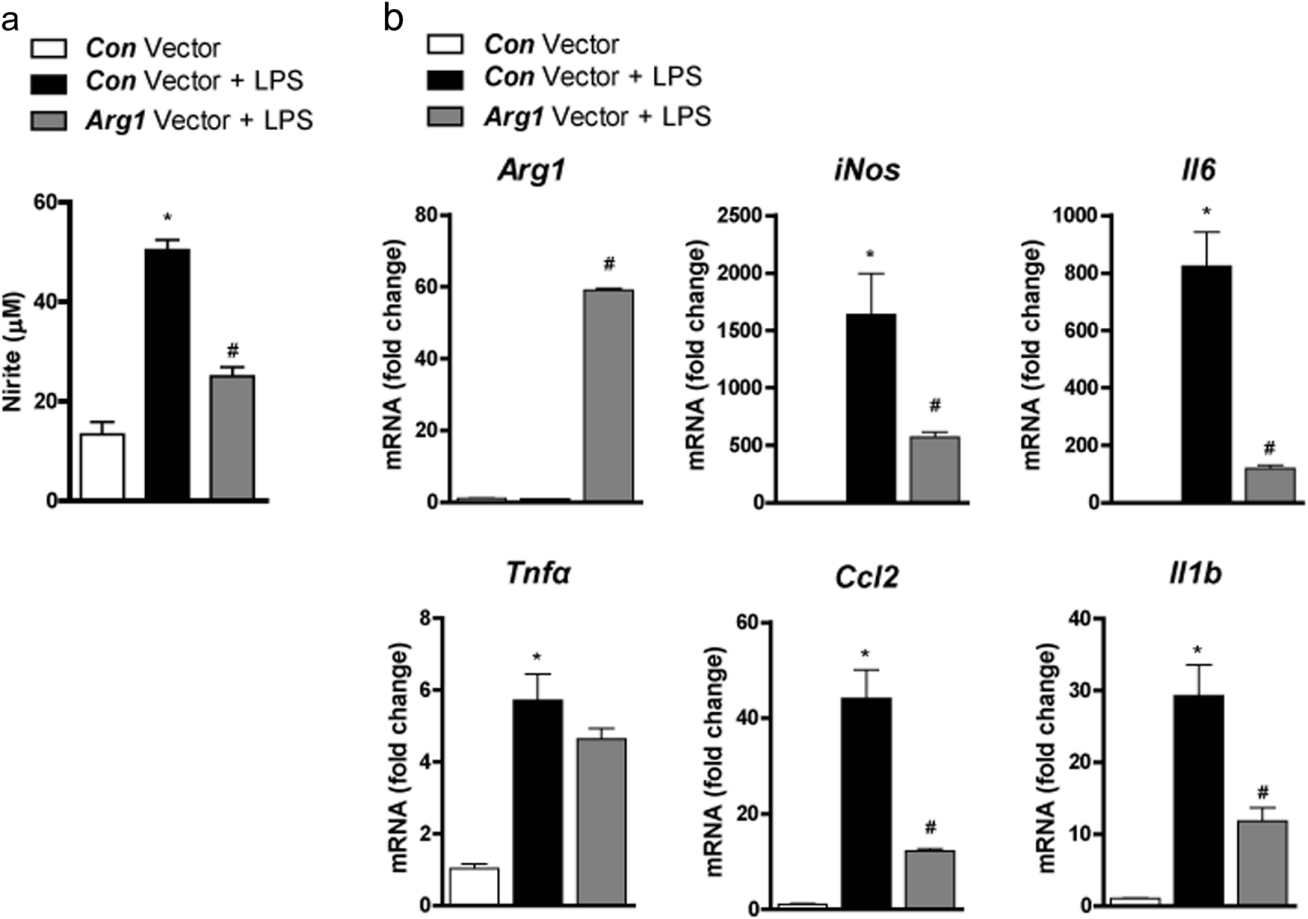
Overexpression of *Arg1* suppressed M1 polarization of macrophages. RAW264.7 cells were transfected with control vector (*Con* vector) or arginase-1 vector (*Arg1* vector) and treated with or without lipopolysaccharides (LPSs). **a** Nitrite concentration in the culture supernatant of RAW264.7 cells after treatment with LPSs for 24 h. **b** Expression of *Arg1* and M1 markers determined using real-time PCR. Data represent means ± SEMs (*n* = 5). ^*^P < 0.05 versus *Con* vector-treated RAW264.7 cells without LPS treatment; ^#^*P* < 0.05 versus Con vector-treated RAW264.7 cells with LPS treatment

As these results suggested that increased *Arg1* expression in macrophages is responsible for the MSC-mediated decrease in M1 activation^27,28^, we next investigated whether *Arg1* overexpression in RAW264.7 cells prevents macrophage activation. For this purpose, RAW264.7 cells were infected with lentivirus overexpressing *Arg1*. As expected^27^, *Arg1-*overexpressing RAW264.7 cells produced less nitric oxide (NO) than the control vector-treated RAW264.7 cells (Fig. 4a). In addition, *Arg1* overexpression decreased the expression of other markers of M1 polarization in RAW264.7 cells (Fig. 4b).

### MSCs reversed cytokine-mediated mitochondrial dysfunction in TECs

Recent studies suggested that inflammatory cytokines produced by activated macrophages impair mitochondrial function in adipocytes^25,26^. Hence, we investigated whether inflammatory cytokines produced by activated macrophages inhibit mitochondrial biogenesis in TECs.

**Fig. 5:**
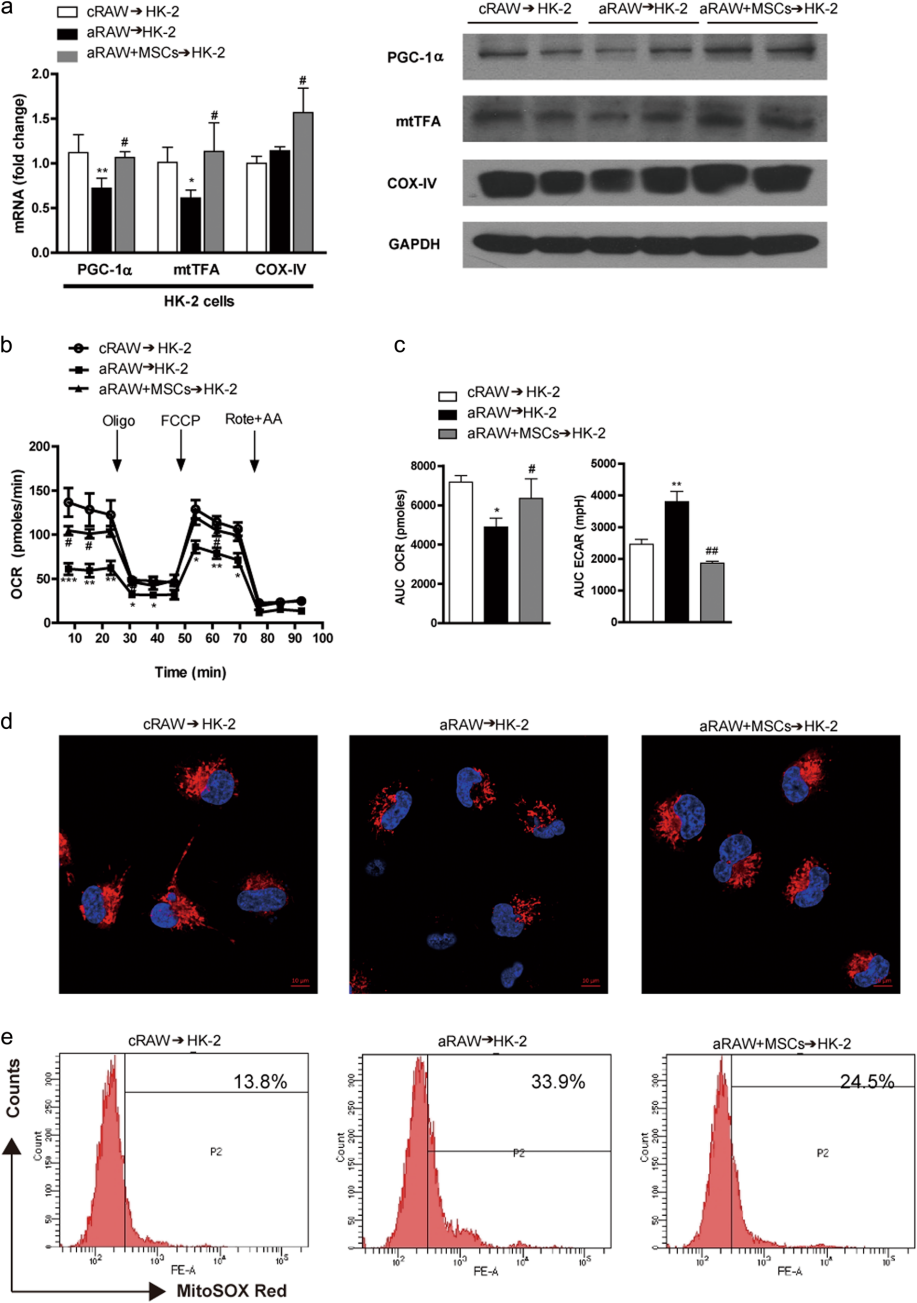
MSCs reversed cytokine-mediated mitochondrial dysfunction in TECs. Conditioned media from RAW264.7 cells (see Fig. 3.) was transferred to HK-2 cells for 24 h. **a** Effect of MSCs on the mRNA (left panel) and protein expression (right panel) of the mitochondrial biogenesis markers PGC-1α, mtTFA, and COX-IV in HK-2 cells. **b** Effect of MSCs on mitochondrial respiration. Real-time OCRs were measured using an XF24 extracellular flux analyzer. During the measurements, 1 μg/ml oligomycin (Oligo), 1 μm carbonyl cyanide p-(trifluoromethoxy)-phenyl-hydrazone (FCCP), and 1 μm rotenone (Rote) plus 2 μm antimycin A (AA) were sequentially added. **c** The area under the curve of the basal OCR (left panel) and the ECAR (right panel). All OCRs and ECARs were normalized based on the cell number. Data show the means ± SEMs (*n* = 5). ^*^P < 0.05, ^**^P < 0.01, and ^***^P < 0.001 versus control (cRAW → HK-2); ^#^*P* < 0.05 and ^##^*P* < 0.01 versus LPS-treated cells (aRAW → HK-2). **d** MSC treatment increased the mitochondrial mass. Mitochondria stained with 100 nm MitoTracker Red are shown in red, and nuclei stained with DAPI are shown in blue. The treatment of HK-2 cells with conditioned media from aRAW cells significantly decreased mitochondrial mass. In contrast, the coculture of MSCs with conditioned media from aRAW cells increased mitochondrial mass. Scale bars, 10 µm (*n* = 4). **e** MSCs decreased mitochondrial ROS production in HK-2 cells, as measured using MitoSOX-based flow cytometry (*n* = 4). cRAW, untreated RAW264.7 cells; aRAW, LPS-treated RAW264.7 cells; aRAW + MSCs, aRAW cells cocultured with MSCs. aRAW → HK-2, HK-2 cells were treated with conditioned media from aRAW cells; aRAW + MSCs → HK-2, HK-2 cells treated with conditioned media from aRAW cells cocultured with MSCs

HK-2 cells, an immortalized proximal TEC line from a normal adult human kidney, were treated with conditioned media from RAW264.7 cells. HK-2 cells treated with conditioned media from aRAW cells (aRAW → HK-2) exhibited decreased *Pgc1a* and mitochondrial transcriptional factor A (*mtTFA)* mRNA levels compared to those in control cells (Fig. 5a). Treatment with conditioned media from aRAW cells cocultured with MSCs (aRAW + MSCs → HK-2) reversed these changes and increased cytochrome C oxidase-IV (*Cox-IV)* expression in HK-2 cells (Fig. 5a). Changes in the protein levels of PGC-1α, mtTFA, and COX-IV were consistent with the observed changes in mRNA expression (Fig. 5a).

We next assessed mitochondrial function in HK-2 cells. Treatment with conditioned media from aRAW cells significantly decreased the OCR and increased the ECAR in HK-2 cells (Fig. 5b, c). The coculture of aRAW cells with MSCs prevented these changes (Fig. 5b, c).

Fluorescence imaging with MitoTracker Red also showed that treatment with the culture media of aRAW cells decreased the mitochondrial mass in HK-2 cells, and coculture of these cells with MSCs reversed this change (Fig. 5d). Increased mitochondrial ROS production, as measured by MitoSOX, is an indicator of mitochondrial superoxide anion (O_2_^−^) production^40^. Flow cytometry showed an increase in the mean fluorescence intensity of MitoSOX in HK-2 cells treated with aRAW-conditioned media, and this was significantly decreased by the coculture of aRAW cells with MSCs (Fig. 5e).

### The beneficial effect of MSCs was limited to umbilical cord blood-derived MSCs

Besides the umbilical cord blood-derived MSCs we used, MSCs can be obtained from bone marrow, adipose tissue, and the placenta. To examine whether this beneficial effect is also shown in other MSCs or limited to MSCs from umbilical cord blood, we tested the effect of AD-MSCs. AD-MSCs exhibited the significantly decreased expression of *Il1b*, *Tnfα*, and *Il6* and increased *Arg1* expression when cocultured with RAW264.7 cells compared to their expression in control cells (Supplementary Fig. 4). However, they also increased the expression of *iNos*, the most important molecule for M1 activity^27^. In addition, AD-MSC supplementation did not reverse mitochondrial dysfunction caused by media from LPS-treated RAW264.7 cells (Supplementary Fig. 4).

### *Arg1* overexpression in macrophages improved mitochondrial function in cultured TECs

**Fig. 6:**
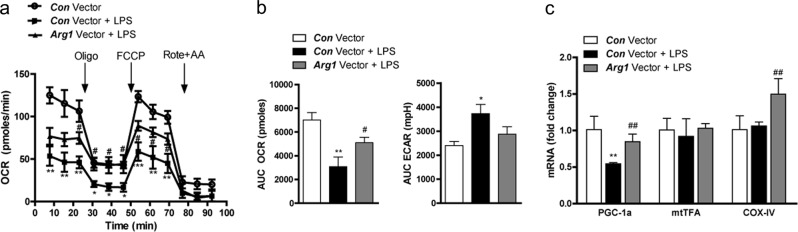
The overexpression of *Arg1* enhanced mitochondrial function in TECs. **a** Real-time OCR rates were measured using an XF24 extracellular flux analyzer. **b** Basal OCRs and ECARs. **c** Relative mRNA levels of mitochondrial biogenesis markers. Data represent the means ± SEMs (*n* = 5). ^*^P < 0.05 and ^**^P < 0.01 versus *Con* vector-treated RAW264.7 cells without LPS treatment; ^#^*P* < 0.05 and ^##^*P* < 0.01 versus Con vector-treated RAW264.7 cells with LPS treatment

Similar to the results obtained from MSC coculture experiments (Fig. 5b, c), HK-2 cells treated with conditioned media from *Arg1*-overexpressing RAW264.7 cells also showed a significantly higher OCR and a lower ECAR than those treated with conditioned media from control vector-expressing RAW264.7 cells (Fig. 6a, b). Consistent with this, treatment with conditioned media from *Arg1*-overexpressing RAW264.7 cells significantly increased the expression of *Pgc1α* and *Cox-IV* in HK-2 cells compared with their expression in untreated cells (Fig. 6c). In contrast, *Arg2* overexpression neither reversed the decrease in *Pgc1α* expression nor increased *Cox-IV* expression in HK-2 cells treated with conditioned media from aRAW cells (aRAW → HK-2) (Supplementary Fig. 5a). Consistent with this, *Arg2* overexpression in macrophages did not affect the expression levels of *Arg1* and *iNos* (Supplementary Fig. 5b).

### IL-10 was not responsible for increased *Arg1* expression in MSCs

Soluble factors produced by MSCs are responsible for alterations in the immune response^41^. Among these soluble factors, IL-10 is known to be the most important immunoregulatory and anti-inflammatory cytokine^41^. However, treatment with recombinant IL-10 did not affect *Arg1* expression (Supplementary Fig. 6), suggesting that *Arg1* induction by MSCs is mediated by soluble factors other than IL-10.

### MSC administration prevented mitochondrial changes in diabetic kidneys

**Fig. 7:**
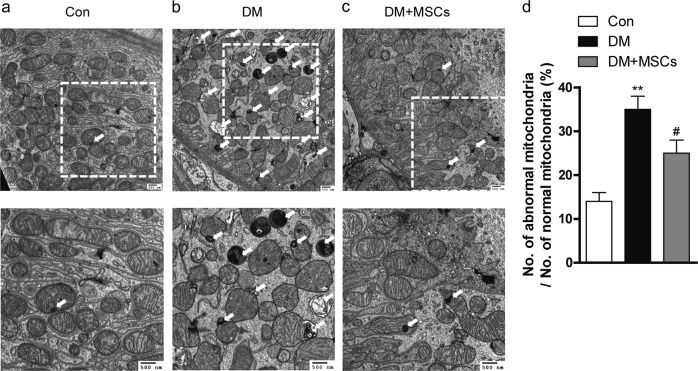
MSC administration prevented mitochondrial changes in the TECs of diabetic kidneys. **a** Representative TEM image of proximal TECs from non-diabetic mice. The normal shape of the mitochondria with a sustained normal pattern of cristae is shown. **b** Representative TEM image of TECs from diabetic mice. Mitochondria with abnormal shapes, such as small, rounded mitochondria and the loss of a normal cristae pattern, were observed. Some mitochondria exhibited an electron-dense structure owing to the aggregation of cristae membranes or focal enlargement of the intermembrane space (white arrows). **c** Representative TEM image of TECs from diabetic mice treated with MSCs. Scale bars, 500 nm. The lower panel is a TEM image at a higher magnification highlighting the region of interest boxed in the upper panel. **d** Quantification of abnormal mitochondria. Data show the means ± SEM, *n* = 20 images/mouse, *n* = 2–3 mice/group. ^**^P *<* 0.01 versus control; ^#^*P* *<* 0.05 versus diabetic mice

We investigated the effect of MSCs on changes in mitochondrial morphology in TECs in vivo. TEM showed swollen mitochondria and distorted cristae of the mitochondria in the TECs of diabetic mice, as described previously^42^, and these changes were ameliorated by MSC treatment (Fig. 7).

## Discussion

In the present study, we observed that treatment with MSCs prevented the progression of diabetic nephropathy by improving mitochondrial function in TECs. MSC treatment decreased the expression of M1 macrophage markers in the kidney and increased *Arg1* expression. Decreased mitochondrial respiration in HK-2 cells, induced by conditioned media from aRAW cells, was abolished when the RAW264.7 cells were cocultured with MSCs or *Arg1* was overexpressed. These observations suggested that MSCs exert their beneficial effects by reversing mitochondrial dysfunction in TECs via the induction of *Arg1* in macrophages.

Mitochondrial dysfunction is a major contributor to the development of diabetic nephropathy^8^. In agreement with this notion, the mitochondria of TECs in diabetic kidneys showed morphological changes including the loss of cristae membranes and a homogenized matrix^42^. Our in vivo and in vitro experiments showed that MSCs improved the mitochondrial function of TECs, which agrees with the results of previous studies on other disease models^11,13^. Paracrine immune modulation by MSCs is an important mechanism for the MSC-mediated rescue and repair of injured organs and tissues^24^. Based on our observations and the results of recent studies showing the regulation of mitochondrial biogenesis by cytokines^25,26^, we suggest that MSCs increase *Arg1* expression in macrophages to reduce M1 macrophage polarization and that this enhances mitochondrial function in TECs.

Besides the umbilical cord blood-derived MSCs we used, MSCs can be obtained from bone marrow, adipose tissue, and the placenta. To examine whether the beneficial effects observed in our study were limited to MSCs from umbilical cord blood, we tested the effect of AD-MSCs. Interestingly, AD-MSCs increased the expression of *iNos* as well as *Arg1*, resulting in a null effect on mitochondrial dysfunction. The reason for this discrepancy between treatment with MSCs from different sources is currently unclear. A recent study reported that umbilical cord-derived MSCs grow faster in vitro and secrete higher concentrations of immunosuppressive cytokines than AD-MSCs^43^. Thus, differences in the secretomes of MSCs from different sources might have different consequences for macrophage polarization, which contributes to mitochondrial function. Future in vivo studies using AD-MSCs are required to clarify their effects on mitochondrial dysfunction in diabetic nephropathy.

Arginase inhibits NO synthesis by competing with iNOS for the same substrate, l-arginine^27,28^. As expected, RAW264.7 cells overexpressing *Arg1* released lower levels of NO than control RAW264.7 cells when treated with LPSs. In addition, *Arg1* overexpression significantly decreased the expression of other M1 macrophage markers, supporting the concept that iNOS and arginase can downregulate each other^27,28^. Although the dominant isoform of arginase in the kidney is arginase-2^37^, in this study, we focused on the effect of MSCs on macrophages. *Arg2* overexpression in macrophages neither increased the expression of *Arg1* nor decreased the expression of *iNos*, suggesting that the effect of MSCs on macrophages is not caused by arginase-2.

Our results agree with those of a recent study showing that the overexpression of *Arg1* significantly reduced inflammatory cytokines in hippocampal tissue^44^. However, this is contrary to the results of another recent study showing that arginase inhibition protected against diabetic nephropathy by increasing endothelial nitric oxide (eNOS) levels in the kidney^45^. It is well known that NO possesses two different cellular activities depending on its concentration^46^. High concentrations of NO are cytotoxic, whereas at low concentrations, NO acts as a signaling molecule that regulates smooth muscle relaxation^46^ and mitochondrial biogenesis^47,48^. iNOS, an enzyme mainly present in macrophages, produces high concentrations of NO and is associated with cytotoxicity, whereas the low concentration of NO produced by eNOS is cytoprotective^48^. Thus, the beneficial effects of arginase in our study resulting from a reduction in the high cytotoxic levels of NO produced by macrophages may be different from the harmful effects that arise from the inhibition of the low physiological levels of NO in the vasculature or kidney parenchymal cells^45^.

**Fig. 8:**
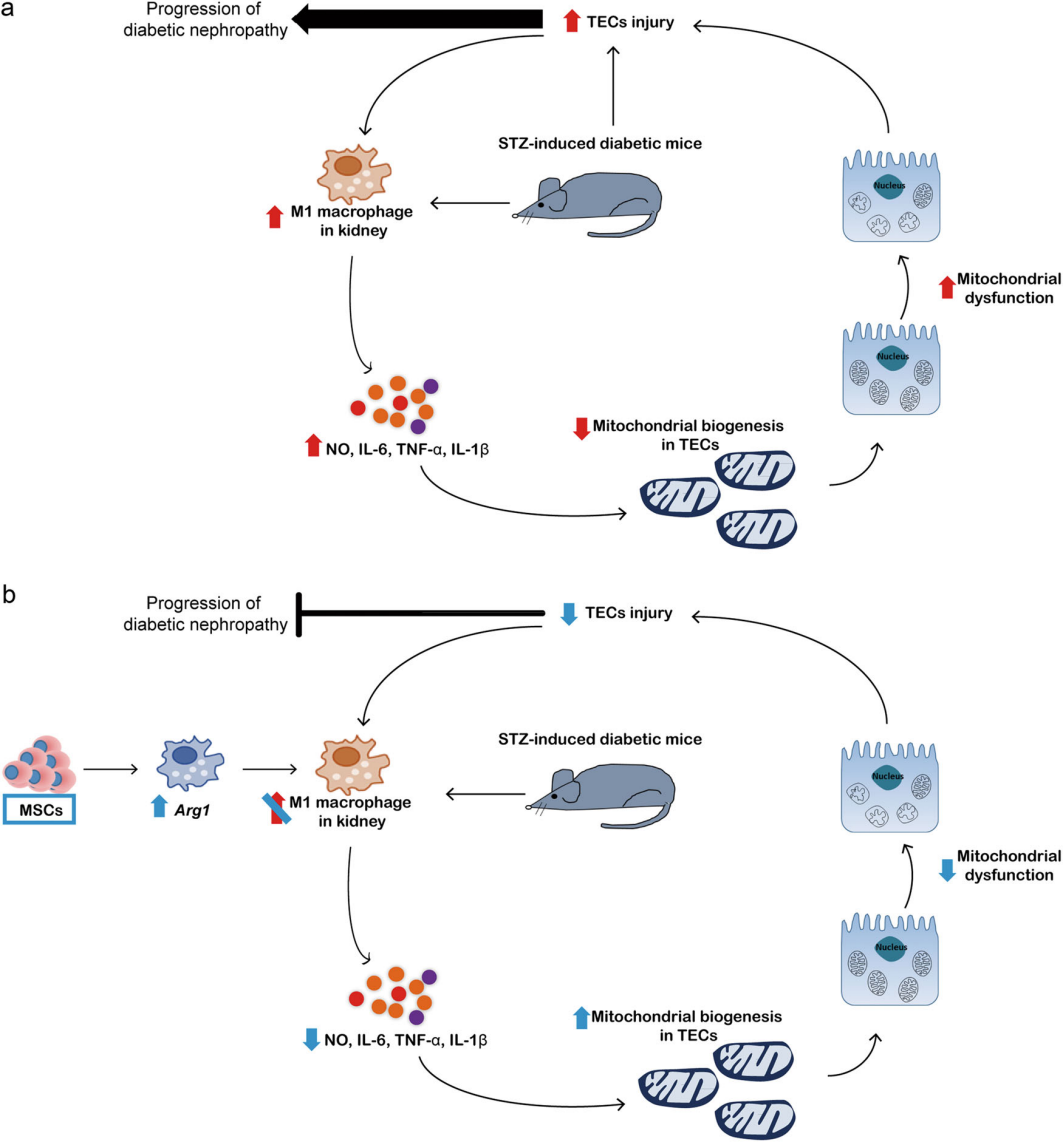
Proposed mechanism by which MSCs prevent the progression of diabetic nephropathy. **a** The M1 macrophage population is increased in diabetic kidneys, and pro-inflammatory cytokines suppress mitochondrial biogenesis to induce mitochondrial dysfunction in TECs. Increased ROS produced from dysfunctional mitochondria cause injury to TECs. In addition, diabetes may lead to the direct injury of TECs. TEC injury may in turn activate M1 macrophages to sustain the progression of diabetic nephropathy. **b** MSCs increase *Arg1* expression in macrophages to suppress M1 macrophage polarization and prevent the deterioration of kidney function

Our study showed that cytokines produced by activated macrophages can induce mitochondrial dysfunction in TECs. On the other hand, a previous study showed that ischemic/reperfusion injury to TECs can recruit M1 macrophages^49^. As mitochondrial dysfunction leads to oxidative stress, cell death, and inflammation^7^, this may also be responsible for secondary tubular cell injury. Taking these results together, we suggest that there may be a vicious cycle of inflammation, mitochondrial dysfunction, injury to TECs, and further inflammation that contributes to the progressive loss of renal function. The MSC-mediated improvement in inflammation and mitochondrial function in TECs may halt this cycle (Fig. 8). Indeed, our study showed that the administration of MSCs decreased UACR and prevented the progression of diabetic nephropathy even after the cessation of MSC therapy.

One of the limitations of our study is that the diabetic mice did not show signs of advanced renal disease, such as significant azotemia or tubulointerstitial fibrosis, in contrast to the results of previous studies^50–53^. The reason for this discrepancy is unknown but may be owing to the different strains used^54^. CD1 mice are outbred, and population substructure and phenotypic differences are observed among CD1 mice obtained from different breeding facilities^54^. Because we used CD1 mice from a Korean company that were different from those used in previous studies^51–53^, differences in the strain might have resulted in the different phenotypes observed in our studies and previous ones. Nevertheless, this does not mean that tubular injury is not important for the development of advanced diabetic lesions. Although pathological analysis of the kidney using light microscopy revealed mainly glomerular lesions, TEM showed mitochondrial damage in the TECs of diabetic mice. In addition, the expression of the markers of tubular injury KIM-1, and NGAL were significantly increased in diabetic mice compared to their expression in control mice*.* In vitro experiments using TECs also demonstrated mitochondrial damage by LPSs and its amelioration by MSCs.

In summary, treatment with MSCs upregulated *Arg1* expression in macrophages and prevented mitochondrial dysfunction in TECs. These effects may halt the vicious cycle of inflammation, mitochondrial dysfunction, injury to TECs, and further inflammation that leads to a progressive decline in kidney function.

## Supplementary information

Supplementary Information
