# Supplementary material for: Mesenchymal stem cells prevent the progression of diabetic nephropathy by improving mitochondrial function in tubular epithelial cells
Source: Exp Mol Med. 2019 Jul 9;51(7):77. doi: 10.1038/s12276-019-0268-5 (PMC6802630; doi:10.1038/s12276-019-0268-5)
Supplement: Supplementary file 1 — Supplementary Information [file 12276_2019_268_MOESM1_ESM.docx]

**Supplementary Table 1. Primer pairs used for real-time PCR analysis**

| **Gene** | **Mouse primer sequences** | |
| --- | --- | --- |
| *αSMA* | Forward | 5'-ACTGGGACGACATGGAAAAG-3' |
|  | Reverse | 5'-GTTCAGTGGTGCCTCTGTCA-3' |
| *Desmin* | Forward | 5'-GTGAGCTTCAGGCCCAGCTT-3' |
|  | Reverse | 5'-TACTGAGCCCGGATGTCCCT-3' |
| *Fn1* | Forward | 5'-GCAAGCCAGTTTCCATCAAT-3' |
|  | Reverse | 5'-CATTTTTGGGAGTGGTGGTCA-3' |
| *Kim-1* | Forward | 5'-ACATATCGTGGAATCACAACGAC-3' |
|  | Reverse | 5'-ACAAGCAGAAGATGGGCATTG-3' |
| *Lcn2* | Forward | 5'-CACAGGTATCCTCAGGTACAGAGCTA-3' |
|  | Reverse | 5'-GGAAAAATACCATGGCGAACTG-3' |
| *Ccl2* | Forward | 5'-TTAAAAACCTGGATCGGAACCAA-3' |
|  | Reverse | 5'-GCATTAGCTTCAGATTTACGGGT-3' |
| *Vcam1* | Forward | 5'-AGTTGGGGATTCGGTTGTTCT-3' |
|  | Reverse | 5'-CCCCTCATTCCTTACCACCC-3' |
| *Icam1* | Forward | 5'-GTGATGCTCAGGTATCCATCCA-3' |
|  | Reverse | 5'-CACAGTTCTCAAAGCACAGCG-3' |
| *Il1b* | Forward | 5'-TCTTTGAAGTTGACGGACCC-3' |
|  | Reverse | 5'-TGAGTGATACTGCCTGCCTG-3' |
| *Tnfα* | Forward | 5'-AGCCCCCAGTCTGTATCCTT-3' |
|  | Reverse | 5'-CTCCCTTTGCAGAACTCAGG-3' |
| *Il6* | Forward | 5'-ACTCCAGAAGACCAGAGGAAAT-3' |
|  | Reverse | 5'-CCAGAGATACAAAGAAATGATGG-3' |
| *iNos* | Forward | 5'-CCAAGCCCTCACCTACTTCC-3' |
|  | Reverse | 5'-CTCTGAGGGCTGACACAAGG-3' |
| *Arg1* | Forward | 5'-GAACCCAACTCTTGGGAAGAC-3' |
|  | Reverse | 5'-GGAGAAGGCGTTTGCTTAGTT-3' |
| *Arg2* | Forward | 5'-TCCTCCACGGGCAAATTCC-3' |
|  | Reverse | 5'-GCTGGACCATATTCCACTCCTA-3' |
| *Tbp1* | Forward | 5'-CCTTCACCAATGACTCCTATGAC-3' |
|  | Reverse | 5'-CAAGTTTACAGCCAAGATTCAC-3' |

| **Gene** | **Human Primer Sequences** | |
| --- | --- | --- |
| *PGC-1α* | Forward | 5'-GCAGTCCTCACAGAGACACT-3' |
|  | Reverse | 5'-AGCCTCATTGTCAGTGGTCA-3' |
| *mtTFA* | Forward | 5'-ATGGCGTTTCTCCGAAGCAT-3' |
|  | Reverse | 5'-ACAAAACTGAAGGGGGAGCG-3' |
| *COX-IV* | Forward | 5'-CAGGGTATTTAGCCTAGTTGGC-3' |
|  | Reverse | 5'-GCCGATCCATATAAGCTGGGA-3' |

**Supplementary Fig. 1. Experimental protocol for MSC therapy in diabetic mice.** Mice were treated with streptozotocin (STZ, 80 mg/kg) dissolved in citrate buffer (pH 4.5) or vehicle for 3 days. Five weeks after induction of diabetes, 5.0 × 10^5^ MSCs or vehicle were intravenously administered thrice every 4 weeks. At 24 weeks after STZ administration, mice were sacrificed and analyzed.

**Supplementary Fig. 2.** **MSCs homing into a kidney.** Eleven weeks after the MSC administration, kidney tissue from diabetic mice was obtained and frozen samples were examined using confocal microscopy. MSCs were stained for human β2-microglobulin. Human β2-microglobulin (green) was merged with DAPI (blue). Original magnification, ×400. Each image is representative of three mice per group.

**Supplementary Fig. 3.** **Summary of cell culture experiments.** RAW cells (cRAW) with or without MSCs were plated and incubated for 24 h, followed by addition of LPS to activate RAW cells (aRAW). 24 hours after LPS treatment, the supernatants were transferred to HK-2 cells for 24 h. Subsequently, the cells were harvested and analyzed.

**Supplementary Fig. 4. The effects of AD-MSCs on macrophage polarization and mitochondrial function.** a: Relative mRNA expression levels of genes representing classically activated M1 macrophages and *Arg1*. b: Relative mRNA expression levels of mitochondrial biogenesis markers including PGC-1α, mtTFA, and COX-IV in HK-2 cells. Data represent means ± SEMs (n = 6). ^*^*P* < 0.05, and ^***^*P* < 0.001 versus control (cRAW); ^#^*P* < 0.05, and ^###^*P* < 0.001 versus LPS-treated RAW cells (aRAW).

**Supplementary Fig. 5. The effects of *Arg2* overexpression on mitochondrial function and macrophage polarization.** a: Relative mRNA expression levels of mitochondrial biogenesis markers in HK-2 cells. b: Relative mRNA expression levels of genes representing classically activated M1 macrophages and *Arg1*. Data represent means ± SEMs (n=4). ^**^*P* < 0.01, and ^***^*P* < 0.001 versus *Con* vector-treated RAW cells without LPS treatment; ^#^*P* < 0.05, and ^##^*P* < 0.01 versus *Con* vector-treated RAW cells with LPS treatment.

**Supplementary Fig. 6. The effects of IL-10 on macrophage polarization.** RAW cells were pretreated with or without IL-10 for 6 h and then incubated with LPS (10 ng/mL) for 24 h. Relative mRNA expression levels of genes representing classically activated M1 macrophages and *Arg1*. Data represent means ± SEMs (n = 6). **P* < 0.05, and ****P* < 0.001 versus control (cRAW).
